# Non-covalent SUMO interactions with (de)conjugation enzymes

**DOI:** 10.1042/EBC20253038

**Published:** 2025-11-04

**Authors:** El Hadji Cisse, Aanchal Mishra, Marcin J. Suskiewicz

**Affiliations:** 1 Centre de Biophysique Moléculaire (CBM), CNRS, Orléans, UPR 4301, France; 2 École Doctorale “Santé, Science Biologique & Chimie du Vivant” (ED549), Université d’Orléans, France

**Keywords:** post-translational modifications (PTMs), SUMO, SUMO E1 SAE1:SAE2, SUMO E2 UBC9, SUMO-specific proteases, SUMOylation

## Abstract

SUMOylation – a protein post-translational modification (PTM) related to ubiquitylation – involves the reversible covalent attachment of the small ubiquitin-like modifier (SUMO) to proteins. During the conjugation and deconjugation cycle, SUMO is recognised and positioned by various enzymes through specific non-covalent interactions. This review discusses the core interactions with the SAE2 subunit of the SUMOspecific heterodimeric E1 enzyme SAE1:SAE2, the SUMO E2 enzyme UBC9 and the SUMO-specific proteases of the SENP family and USPL1. We describe the evolutionary origins of these interactions and their structural basis; moreover, as SUMO:enzyme interactions are generally similar in their overall outline to those between ubiquitin and its specific enzymes, we highlight these similarities, as well as the differences. All of the mentioned interactions use a similar surface on SUMO, which is distinct from the groove that binds SUMO-interacting motifs (SIMs), meaning that while the enzyme interactions are mutually exclusive, each is compatible with simultaneous SIM binding. This review is accompanied by another in the same issue that focuses on interactions with SUMO E3 ligases and downstream effectors of SUMOylation, together providing comprehensive coverage of the non-covalent interactions formed by SUMO proteins.

## Introduction

Enzymatic protein post-translational modifications (PTMs) – added and removed by dedicated enzymes – are critical modulators of protein half-life, interaction networks, localisation and overall function [1]. Among these, SUMOylation – the covalent attachment of small ubiquitin-like modifier (SUMO) to proteins – is an essential PTM in eukaryotes, playing a central role in numerous cellular processes such as transcription, nuclear transport and DNA repair [2–5].

**Figure 1:**
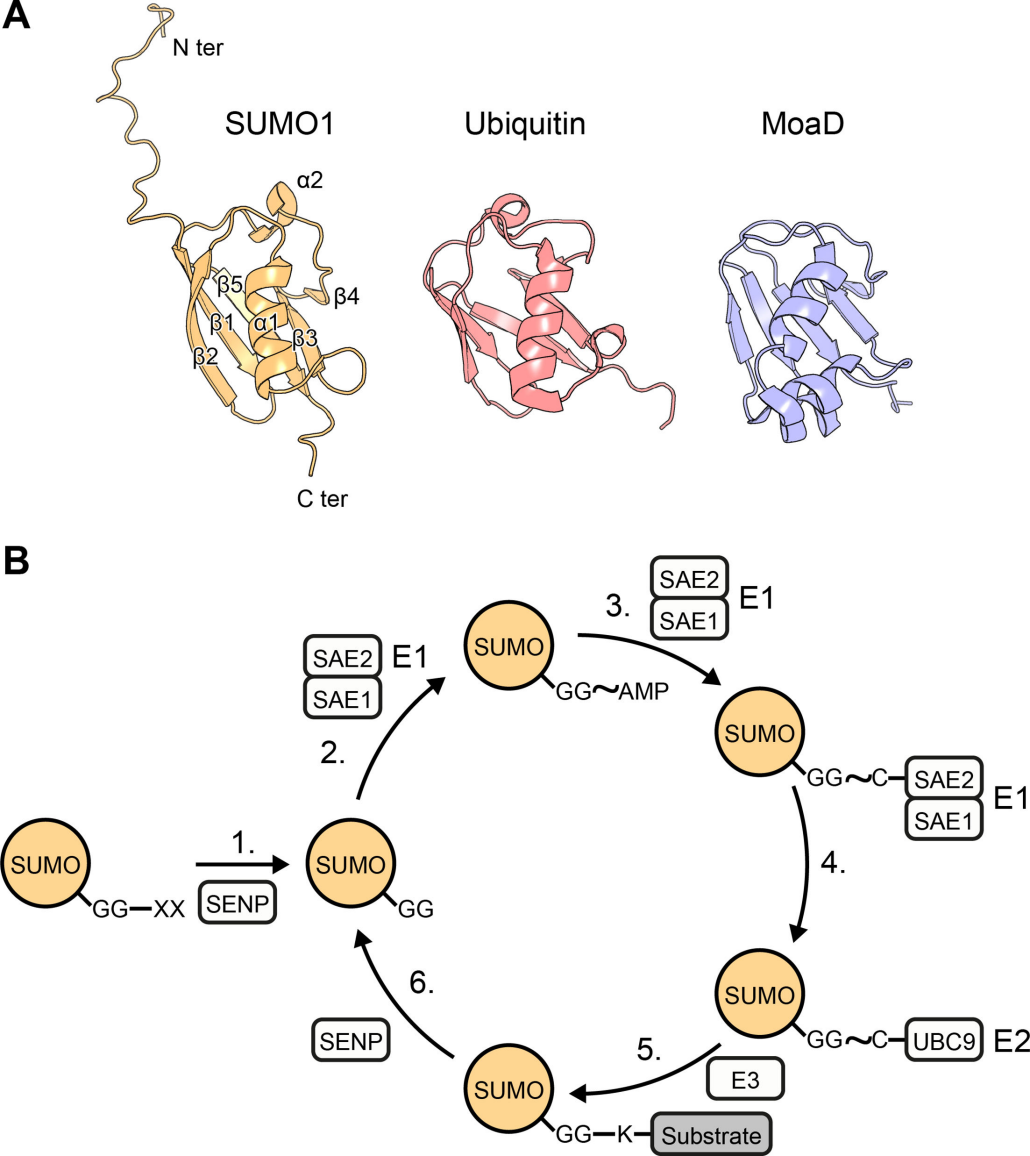
SUMO structure and (de)conjugation cycle **A.** Molecular structure of mature human SUMO1 (residues 2–97 from PDB 1A5R, light orange), human ubiquitin (PDB 1D3Z, pink) and prokaryotic sulphur-carrier protein MoaD (PDB 1FM0, chain D, blue). N- and C-termini and secondary structural elements within SUMO1 are labelled. **B**. The SUMO (de)conjugation cycle composed of maturation of SUMO precursors by SENP proteases (step 1); a conjugation cascade (steps 2–5) comprising E1-catalysed adenylation with ATP (step 2), transfer to the catalytic Cys of the SAE2 subunit in E1 (step 3), transthioesterification from the catalytic Cys of SAE2 to the catalytic Cys of the E2 enzyme UBC9 (step 4) and E3-dependent or -independent transfer of SUMO from UBC9 to a Lys residue on the substrate (step 5); followed by SENP-catalysed deconjugation (step 6).

SUMO, a homologue of ubiquitin (Figure 1A), is conjugated to substrates through a dedicated enzymatic cascade similar to – but simpler than – that of ubiquitin, involving a single E1 enzyme, a single E2 enzyme and a limited number of E3 ligases [6,7] (Figure 1B).

During this cascade, SUMO forms transient covalent anhydride and thioester attachments, which ultimately give way to a chemically stable (but enzymatically cleavable) isopeptide bond with a target Lys residue on a substrate. The SUMOylation cycle is completed by a deconjugation step catalysed by SUMO-specific proteases, the reaction proceeding via a transient covalent tetrahedral intermediate. The same enzymes also perform proteolytic maturation of SUMO prior to its use as a modifier.

Beyond the stable and transient covalent bonds formed during the catalytic cycle, the function of SUMO depends on its non-covalent interactions, some of which again are integral to the (de)conjugation process itself. These include interactions with catalytic domains or regions of E1, E2 and E3 enzymes or SUMO-specific proteases that help recruit and position SUMO for the respective reactions. In addition, the functional reach of SUMO is vastly extended through non-covalent interactions with downstream effector proteins.

While interactions with SUMO E3 ligases and effectors tend to involve short linear motifs known as SUMO-interacting motifs (SIMs) [8–10], core interactions with E1, E2 and protease enzymes rely on extended interaction surfaces on catalytic domains of these proteins. These enzymes contact partly overlapping surface areas on the SUMO β-sheet, which are distinct from the groove that binds SIMs.

In this review, we discuss the properties of SUMO as a covalent modifier and a non-covalent interaction hub and provide an overview of its interactions with key enzymes of the (de)conjugation cycle, including E1, E2 and SUMO-specific protease enzymes (but excluding SUMO E3 ligases). We discuss the structural basis, evolution and, briefly, the functions of these interactions. Interactions with SUMO E3 ligases and effectors are covered in an accompanying review. Throughout the text, SUMO residue positions are referred to using human SUMO1 numbering unless stated otherwise. We use ‘–’ for chemically stable covalent bonds, ‘~’ for labile covalent bonds, and ‘:’ for non-covalent complexes.

## SUMO proteins and their features relevant to non-covalent interactions

In this section, we will briefly introduce the early discoveries that laid the foundation of the SUMOylation field and present the SUMO proteins, focusing on the aspects that are relevant to understanding their non-covalent interactions – including their evolution, structure, dynamics and surface properties.

### Early history of SUMOylation research

The earliest studies pertaining to SUMOylation, carried out mainly with yeast, human and *Xenopus* systems, emerged in the period of extraordinary productivity by several labs in the mid to late 1990 s. The SUMO proteins/genes were first encountered in a genetic screen for suppressors of mutations in a centromeric protein in yeast [11] and in yeast two-hybrid screens for human interactors of human DNA-repair proteins RAD51 and RAD52 [12], cell death-related receptors FAS and TNFR1 [13], and the protein PML, which is known to get fused to RARA in a specific type of leukaemia [14].

Although all these studies noted the homology between the newly identified protein and ubiquitin – which was by then well established as a protein modifier – it might have appeared that SUMO acts mainly through non-covalent interactions with other proteins. However, the work from the laboratories of Günter Blobel (performed by Michael Matunis, Erica Johnson and colleagues) [15,16], Larry Gerace (led by Frauke Melchior) [17] and Mary Dasso (spearheaded by Hisato Saitoh) [18,19] soon showed that SUMO can also be covalently attached to proteins as a *bona fide* PTM. The first identified SUMO target was RANGAP1, a key regulator of nucleocytoplasmic transport, still used as a model substrate due to its exceptionally high SUMOylation efficiency.

These discoveries were quickly followed by the identification of the enzymes necessary for SUMO conjugation, with a key contribution from Ronald Hay’s, Günter Blobel’s and Stefan Jentsch’s laboratories [16,20–24]. These enzymes turned out to be evolutionarily related to those catalysing ubiquitylation, yet specific for SUMO. Around the same time, different genes for SUMO paralogues were also identified [25,26]. A critical role in the subsequent mechanistic elucidation of SUMOylation mechanisms – including the non-covalent interactions with the enzymes discussed here – has been played by structural biology, with a key contribution by Christopher D. Lima’s laboratory (e.g. [27–31]).

### Natural history of SUMO as a ubiquitin-like protein

SUMO proteins are part of a broader family of ubiquitin-like (UBL) modifiers. Both the emergence of the first UBL modifier and its divergence into ubiquitin and SUMO probably predate the origin of eukaryotes [32,33] (Figure 1A). Some aspects of eukaryotic UBL modifier biochemistry – notably its characteristic β-grasp fold and the ability to be activated by an E1-like adenylation (AMPylation) step – are shared with prokaryotic sulphur carrier proteins, such as MoaD and ThiS, which do not become conjugated to protein substrates and probably represent a more ancestral function of UBL systems [32,34–36]. The β-grasp fold itself dates even further back, possibly to primitive RNA-binding proteins [37].

In eukaryotes, in addition to UBL modifiers – often referred to as type-I UBL proteins and including ubiquitin, SUMO, ATG8/LC3, NEDD8, ISG15 and others – we find multidomain proteins with ubiquitin-fold domains that are not conjugated to substrates, classified as type-II UBL proteins [38]. This review focuses on the non-covalent interactions of SUMO and, to a small extent, of other type-I UBL proteins, insofar as these resemble, or differ from, SUMO interactions. Nonetheless, of some interest to our discussion are also type-II UBL proteins with SUMO-like domains (described as such – rather than simply UBL domains – due to their closer similarity to SUMO compared with ubiquitin), especially the deubiquitinase UAF1, whose SUMO-like domain binds to a SIM-like loop region on its substrate FANCI [39], and human NFATC2IP/NIP45 and its yeast counterpart ESC2, whose SUMO-like domains apparently bind to the back of SUMO E2 enzyme [40,41].

### SUMO orthologues and paralogues

Broadly present in eukaryotes, SUMO was likely present in their last common ancestor [33]. In the model yeast *Saccharomyces cerevisiae*, there is only a single SUMO orthologue called Smt3. However, in more complex metazoans and plants, SUMO has repeatedly undergone duplication and diversification into multiple, sometimes quite different, paralogues [42].

To date, five distinct SUMO paralogues have been identified in humans: SUMO1 to SUMO5 [25,26,43], each encoded by a separate gene. Unlike ubiquitin, which is partly expressed as head-to-tail fusions of multiple copies that must be cleaved into individual units, each SUMO paralogue is expressed as a single, separate protein – albeit with a few extra C-terminal residues that must be removed during SUMO maturation to expose the C-terminal Gly–Gly motif needed for conjugation. Furthermore, as the initiator Met residues of SUMO paralogues are followed by Ala or Ser, they are cleaved off in the cell.

The diversity of SUMO signals is increased by the fact that SUMO can be attached to substrates not only as a single unit (monoSUMOylation) at a single site but also singly at multiple sites (multiSUMOylation) or as chains of SUMO molecules linked through isopeptide bonds (polySUMOylation).

**Figure 2:**
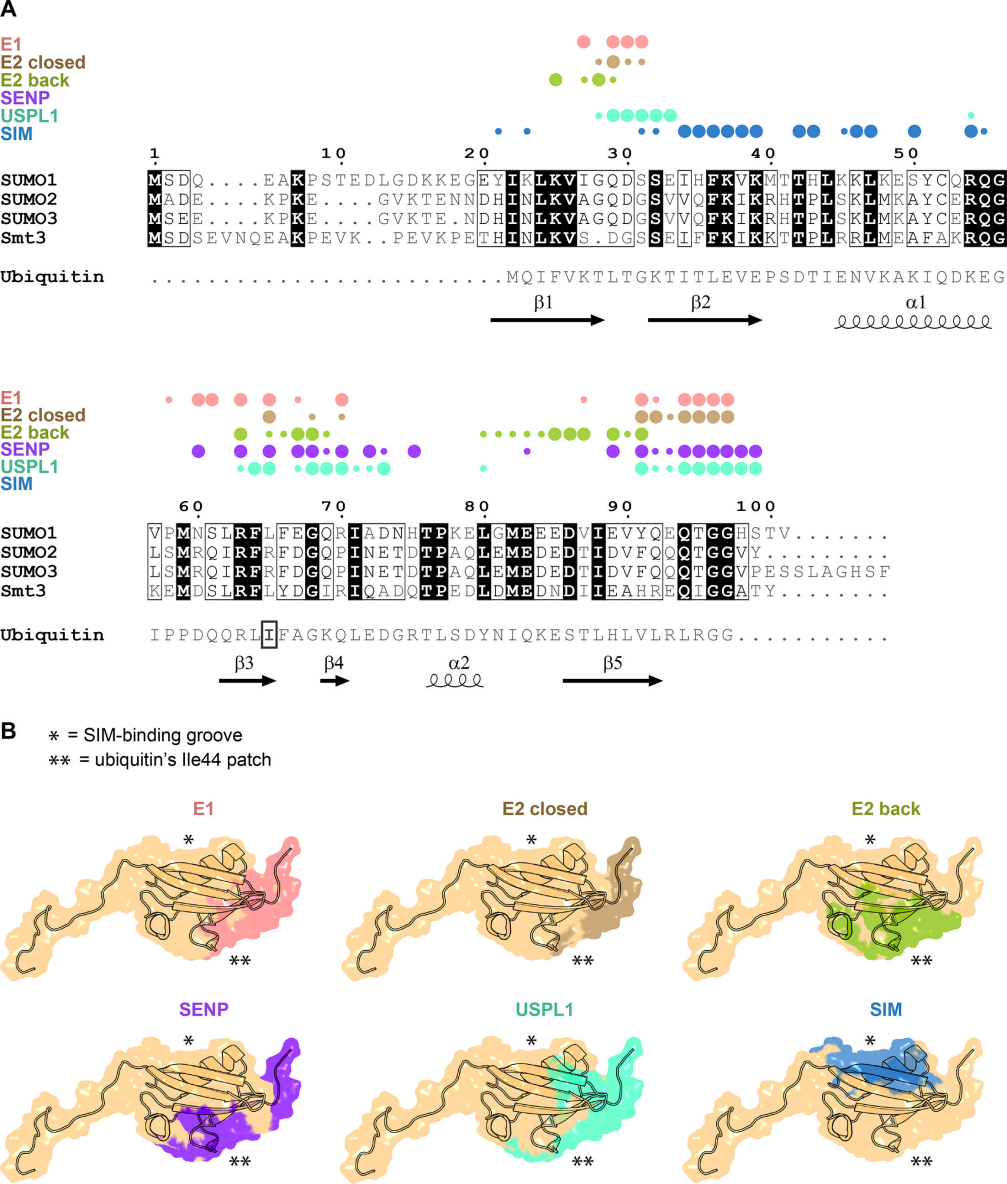
SUMO residues and surfaces mediating interactions with enzymes and SIMs **A.** Multiple sequence alignment of sequences of human SUMO1, SUMO2 and SUMO3 and *saccharomyces cerevisiae* Smt3. Aligned human ubiquitin sequence is provided underneath for reference, with its Ile44 residues marked with a rectangle. The alignment was performed with Clustal Omega with default settings, processed using ESPript 3.0 [45] and further manually adapted. Residue numbering and secondary structure assignment corresponds to human SUMO1. Above the alignment, SUMO residues buried by the following interactions are indicated with coloured circles: SUMO bound to SAE2 subunit of the E1 enzyme (‘E1’, pink); donor SUMO that is part of SUMO∼UBC9 thioester in the closed conformation, interacting with UBC9 (‘E2 closed’, orange); SUMO non-covalently bound at the back of UBC9 (‘E2 back’, green); SUMO bound to SUMO-specific protease SENP2 (‘SENP’, violet); and SUMO bound by a SUMO-interacting motif (SIM) from RANBP2 (‘SIM’, blue). The residues were identified with PISA Server using PDB entries 1Y8R for E1, 2PE6 for UBC9 back, 3UIP for UBC9 closed and SIM and 1TGZ for SENP. Small circles indicate only partial burying of a given residue by the indicated interaction. **B**. A molecular structure of human SUMO1 (residues 2–97 from PDB 1A5R, light orange) in transparent space-filling surface representation with the interactions from point A indicated as coloured patches. The approximate locations of the SIM-binding groove or the Ile44 patch of ubiquitin are indicated with asterisks, as explained in the figure.

SUMO1, SUMO2 and SUMO3 appear to be the most broadly expressed and used SUMO paralogues [44]. SUMO2 and SUMO3 are virtually identical in amino acid sequence (98% identical over the length of the mature protein) and are often jointly referred to as SUMO2/3, whereas SUMO1 is significantly different (47–48% identical over the same region) (Figure 2A). The limited identity between SUMO2/3 and SUMO1 allows a degree of paralogue specificity – particularly among SUMO proteases and readers – but begs the question of how these different paralogues can utilise the same core enzymatic machinery. Two more rarely expressed SUMO paralogues, SUMO4 and SUMO5, are closely similar (85–88% identity) to SUMO2/3 and SUMO1, respectively, but whether SUMO4 can be conjugated to substrates remains controversial, as its Gly–Gly motif is preceded by a proline two residues upstream, which hinders proteolytic maturation [43,46–49]. SUMO4 might instead function mainly via non-covalent interactions, through which it apparently enhances SENP1’s deconjugation activity [49]. Despite the differences, SUMO paralogues are still considerably more similar to each other than they are to ubiquitin (only 16-18% identity), consistent with the orthogonality of the SUMO and ubiquitin conjugation and deconjugation machineries.

### SUMO structure and dynamics

Structurally, SUMO has retained the β-grasp fold of ancestral UBL modifier and prokaryotic sulphur-carrier proteins (Figure 1A). This fold is named for a β-sheet that wraps around a central α-helix, resembling a ‘molecular handshake’. The mixed SUMO β-sheet is arranged in the order β2:β1:β5:β3:β4, with β2 and β3 running antiparallel to the adjacent strands [50–52]. The α1 helix lies diagonally across the concave face of the β-sheet, forming a hydrophobic core that stabilises the overall architecture. See the assignment under the multiple sequence alignment in Figure 2A for the residues corresponding to each secondary-structure element.

The compact fold of SUMO’s core masks its dynamic nature, with motions revealed by NMR relaxation experiments – particularly in loops and other functionally important regions, including the SIM-binding groove [53,54]. These dynamic features – which might differ between paralogues and need to be further investigated – could mediate allosteric signals and facilitate molecular recognition by allowing SUMO to adapt its surface to different binding partners.

### SUMO surface properties

Notably, SUMO contains a specialised hydrophobic groove for binding specific SIM motifs, located at the exposed edge of its β-sheet, between the β2 strand and the α1 helix. The groove is surrounded by conserved hydrophobic residues such as Ile33, Phe36, Val38 and Leu45 and, at one end, positively charged residues, notably Lys39 and Lys46. A hydrophobic groove at this location is missing or less developed in ubiquitin, but similar features can be found in UBL modifiers of the ATG8/LC3 family [55], UFM1 [56] and ATG12 [57], where they have also been observed to bind linear motifs adopting at least partly β-strand conformations. Some or all of these modifiers might have inherited the groove from their common ancestor.

Other than the hydrophobic groove, the surface of SUMO is relatively polar and charged, with a positively charged patch around the mentioned Lys39 and Lys46 residues (including also Lys78 and His43), and a pronounced negatively charged patch composed of Asp or Glu residues in positions 67, 83–86 and 89.

Importantly, SUMO lacks a clear hydrophobic patch equivalent to the Ile44-centred surface of ubiquitin at the opposite side of the β-sheet relative to the SIM-binding groove. Ile44 itself is only partially conserved as Leu65 in SUMO1 and yeast Smt3, and replaced by an Arg in SUMO2/3 (Figure 2A). Moreover, the surrounding surface is generally less hydrophobic in all SUMO paralogues. Nonetheless, this surface mediates important SUMO interactions, notably with conjugation and deconjugation enzymes, as discussed below.

### SUMO interaction surfaces

A useful classification of SUMO non-covalent interactions divides them according to the region of SUMO involved in the binding into class I (SIM-binding groove-dependent, explored in the accompanying review), class II (involving the opposite side of the SUMO β-sheet overlapping with ubiquitin’s Ile44 patch and discussed in this review) and class III (apparently mediated by yet another SUMO surface near its N-terminal tail and less explored – see the section about ZZ domains in the accompanying review) interactions [7].

The two most important SUMO surfaces in terms of the number of known non-covalent interactions are the SIM-binding groove and the opposite side of the SUMO β-sheet. Both appear essential, as reflected in the lethal phenotypes of certain point mutants targeting either surface in yeast *S. cerevisiae* [58]. The surface opposite the SIM-binding groove might be more ancient, as it mediates critical interactions with E1 enzymes and the back of E2 that are conserved in their overall outline among UBL modifier systems. However, while in ubiquitin this surface – often referred to as the Ile44 patch – mediates most non-covalent interactions, both with enzymes and with the majority of downstream effectors [59], in SUMO it primarily mediates enzyme interactions and appears less prone to engage in additional interactions. Indeed, in SUMO, the role of the Ile44 patch as a ‘sticky’ epitope for which new non-covalent binders readily evolve appears to have been taken over by the SIM-binding groove. This difference is well illustrated by results obtained during artificial affimer selection: those selected using ubiquitin target the Ile44 patch [60], whereas those selected for SUMO binding feature a SIM-like β-strand that engages the SIM-binding groove [61].

### SUMO termini

The compact structure of the SUMO core is flanked by flexible N- and C-terminal tails (Figure 1A). The N-terminal tail, comprising approximately the first 15–17 amino acids, is likely largely intrinsically disordered, although part of it may have a helical character under specific conditions – as observed in human SUMO1 in the presence of a phosphorylated SIM and zinc ions [62]. This flexible region appears to dynamically interact with the SIM-binding groove, thereby partially inhibiting SIM binding [62,63], as discussed in more detail in the accompanying review. Additionally, the N-terminal tail serves as the primary site for SUMO chain elongation [64,65]. Finally, the extreme N-terminal residues have been shown to interact with the ZZ domains of HERC2 [66], as also discussed in the accompanying review. However, despite these functions, the N-terminal region appears dispensable for the core SUMO functions, at least in yeast, as reflected by the lack of phenotype observed upon its deletion in *S. cerevisiae* under the tested growth conditions [58].

The C-terminal tail, although shorter than its N-terminal counterpart, also appears to be largely flexible in the unbound state. It serves as the site of SUMO maturation by SUMO-specific proteases and is also contacted by the other enzymes, including E1 and E2. When bound to SENP proteases, a short segment of this tail adopts a β-strand conformation [12,27,30,67], whereas in the other enzyme-bound states, it appears to adopt an ordered but more irregular conformation [28,68]. The C-terminal sequence upstream of the Gly–Gly motif differs markedly between SUMO and ubiquitin (-QEQTGG in SUMO1 vs -LRLRGG in ubiquitin, see Figure 2A), which serves as one of the determinants that allow SUMO-specific enzymes to discriminate against ubiquitin. The sequence downstream of the Gly–Gly motif is different between SUMO paralogues (-GGHSTV in SUMO1, -GGVY in SUMO2 and -GGVPESSLAGHSF in SUMO3), apparently translating into differential recognition of the immature forms of SUMO paralogues by SUMO-specific proteases [69–71]. SUMO proteases can also detect slight differences between SUMO paralogues upstream of the Gly–Gly motif [71].

The inherent flexibility of the SUMO C-terminus implies that the SUMO∼E1 and SUMO∼E2 thioester intermediates do not exhibit a fixed orientation of SUMO relative to the enzyme, unless stabilised by additional contacts – an insight with implications for the mechanism of SUMOylation, as discussed below. Similarly, the final SUMO–substrate conjugate is also unlikely to be rigid unless stabilised by additional interactions between the substrate and the SUMO core, as observed in at least one SUMOylated substrate protein, TDG [72].

## The SUMOylation cycle

SUMO is conjugated to substrates through a conserved enzymatic cascade, which is homologous to that for ubiquitin and has been described in more detail elsewhere (see [31] and [73] for reviews discussing conjugation cascades of UBL proteins in general, and [6] and [7] specifically of SUMO). SUMO modification can be removed through enzymatic deconjugation, resulting in the SUMOylation cycle, discussed briefly below (Figure 1B).

### Evolutionary origins of UBL conjugation cascades

From an evolutionary perspective, the earliest components of the UBL conjugation system appeared in prokaryotes and consisted of the UBL protein itself alongside an E1-like enzyme capable of attaching AMP to its C-terminus – initially for functions other than activating the UBL for protein modification [32,36]. Extant examples of such prokaryotic E1-like enzymes include MoeB and ThiF, which adenylate their respective UBL partners, MoaD and ThiS, without promoting their conjugation to other proteins [34,35]. Instead, these proteins act as sulphur carriers.

Still in prokaryotes, the ancestral ubiquitylation cascade evolved further, enabling transfer of a UBL protein from AMP to Cys residues in E1 and E2 enzymes, and ultimately to a Lys residue on protein substrates. Prokaryotic UBL conjugation systems were first predicted using bioinformatics [36,74,75] and then validated and characterised experimentally, first in archaea [76] and recently in bacteria [77–82]. Several recent articles point to a relative abundance and diversity of bacterial operons coding for UBL (de)conjugation cascades, in some cases – such as those of the *Bli* and *Bub* operons – complete with a UBL protein, E1 and E2 enzymes, and a specific protease [79–81]. These systems appear to function primarily in the defence against bacterial viruses known as phages and might have proliferated under infection pressure, consistent with the notion that PTMs had generally emerged in contexts of biological conflicts [83]. Structurally, as observed by Chambers et al., the bacterial UBL modifiers more closely resemble eukaryotic ubiquitin than bacterial sulphur carrier proteins such as ThiS and MoaD [80]. Overall, these observations support a hypothesis that the origins of ubiquitylation and related pathways can be traced back to bacteria. In eukaryotes, the ancestral conjugation cascades have eventually diverged into multiple orthogonal UBL systems simultaneously operating within a single cell, complete with their respective E1, E2 and E3 enzymes.

### The SUMOylation cascade

Following their proteolytic maturation by SUMO-specific proteases, which exposes the C-terminal Gly–Gly motif, SUMO proteins are first adenylated (AMPylated) by the SUMO E1 activating enzyme, a heterodimer composed of SAE1 and SAE2 subunits. In this step, an AMP moiety derived from ATP is covalently attached to SUMO’s Gly–Gly motif via a labile mixed anhydride bond, accompanied by the subsequent release of pyrophosphate.

The resultant SUMO adenylate is an activated form of SUMO, with the AMP adduct having features of a good leaving group that can be efficiently replaced by another nucleophile, an active-site Cys residue (Cys173 in humans) of the SAE2 subunit of E1. This reaction results in the formation of a SUMO∼SAE2 thioester intermediate and release of AMP. This is then followed by a second nucleophilic attack, by a Cys residue (Cys93) of the E2 enzyme, UBC9, leading to transthioesterification and the formation of a SUMO∼UBC9 thioester intermediate [16,19,22]. From there, SUMO is eventually moved to a target Lys residue. Overall, this pathway can be summarised as SUMO being C-terminally activated and relayed between different reactive groups before ultimately ‘landing’ on a Lys residue of a substrate. Each transfer of SUMO between groups is expected to occur via an addition–elimination mechanism involving a tetrahedral intermediate.

Although UBC9 alone is capable of directly discharging SUMO onto Lys residues in substrates, and is apparently uniquely capable among UBL E2 enzymes of directly recognising certain consensus motifs (notably the so-called SUMOylation consensus motif, with a hydrophobic residue preceding Lys and an acidic residue two residues after Lys [84,85]), the efficiency and specificity of substrate SUMOylation can be enhanced by SUMO E3 ligases [7,68,86–88]. E3 ligases are important in both SUMOylation and ubiquitylation, but they play a more critical role in ubiquitylation, where they are often the main, if not the sole, determinant of substrate specificity [89].

### SUMO deconjugation

Whereas all UBL E1 and E2 enzymes are homologous to their counterparts from other UBL pathways – suggesting that the core E1–E2 cascade emerged once and subsequently diversified through divergent evolution – the UBL deconjugation function appears to have evolved independently multiple times, arising in many different protease scaffolds. This is particularly striking in the case of ubiquitin proteases (also called deubiquitylating enzymes or DUBs), several distinct types of which have been identified [90].

For SUMO, at least three eukaryotic SUMO-specific protease families have been described [91]. The main class, known as SENPs (sentrin/SUMO-specific proteases, sentrin being one of SUMO’s early names) in vertebrates and ULPs (ubiquitin-like proteases) in yeast, belongs to the C48 family of Cys proteases [91,92]. In mammals, six SUMO-specific SENPs have been identified (SENP1-3 and SENP5-7; SENP4 is identical to SENP3). Although SENP8 (also called DEN1) shares structural similarity with these enzymes, it acts on a different UBL, NEDD8 [93–97]. Some SENPs, including SENP1, SENP2 and SENP5, are involved in both processing SUMO precursors and removing SUMO from substrates [92]. In contrast, SENP6 and SENP7 appear to be limited in their ability to carry out SUMO maturation, instead specialising in cleaving polySUMO chains [97]. Moreover, individual SENPs have partially distinct preferences for SUMO paralogues and, possibly, substrate proteins [92].

The catalytic mechanism of SUMO deconjugation and maturation by SENPs involves *trans*-to-*cis* isomerisation of the (iso)peptide bond, providing a geometry favourable for nucleophilic attack by the thiol group of their catalytic cysteine [30,67]. This step, in turn, leads to the formation of an unstable tetrahedral intermediate, culminating in (iso)peptide-bond cleavage.

While SENPs/ULPs remain the canonical and best-characterised SUMO proteases, additional non-SENP enzymes with SUMO deconjugation activity have been reported. These include DESI1 and DESI2, members of the PPPDE family of permutated papain-like Cys proteases [98], and USPL, which belongs to the ubiquitin-specific protease (USP) family of canonical (non-permutated) papain-like Cys proteases [71,99]. Despite its classification alongside DUBs, USPL1 lacks efficient deubiquitylating activity and appears functionally specialised for SUMO removal. Interestingly, all confirmed and suggested SUMO-specific proteases seem to utilise a Cys-dependent mechanism.

Although UBL proteases are generally specific to a given UBL, some natural proteases have broader activity affecting multiple UBLs. This is the case for NopD from *Bradyrhizobium* sp. XS1150 and XopD, a type-III effector protein secreted by the phytopathogenic bacterium *Xanthomonas campestris* pathovar vesicatoria (Xcv), both of which belong to the C48 family of cysteine proteases and are able to cleave several different UBLs, including both SUMO and ubiquitin [100–103].

## Non-covalent SUMO:E1 interactions

The SUMO E1 enzyme inherited its basic adenylation mechanism from the ancestral UBL E1 enzyme and, ultimately, from prokaryotic E1-like enzymes acting on sulphur carrier proteins [104]. Along with the mechanism itself, SUMO retained the ancestral non-covalent interaction between the adenylation domain and the UBL protein, which helps to correctly position the UBL protein for the reaction with ATP.

Prokaryotic E1-like enzymes such as MoeB or ThiF comprise only an active adenylation domain and form homodimers; in contrast, eukaryotic E1 enzymes are generally heterodimers consisting of two homologous subunits with a pseudo-symmetrical organisation comprising one active and one inactive adenylation domain (sometimes fused into a single monomeric polypeptide, as in the case of the human ubiquitin-specific E1, UBA1), as well as additional domains that catalyse E1 and E2 thioester formation following adenylation [104].

**Figure 3:**
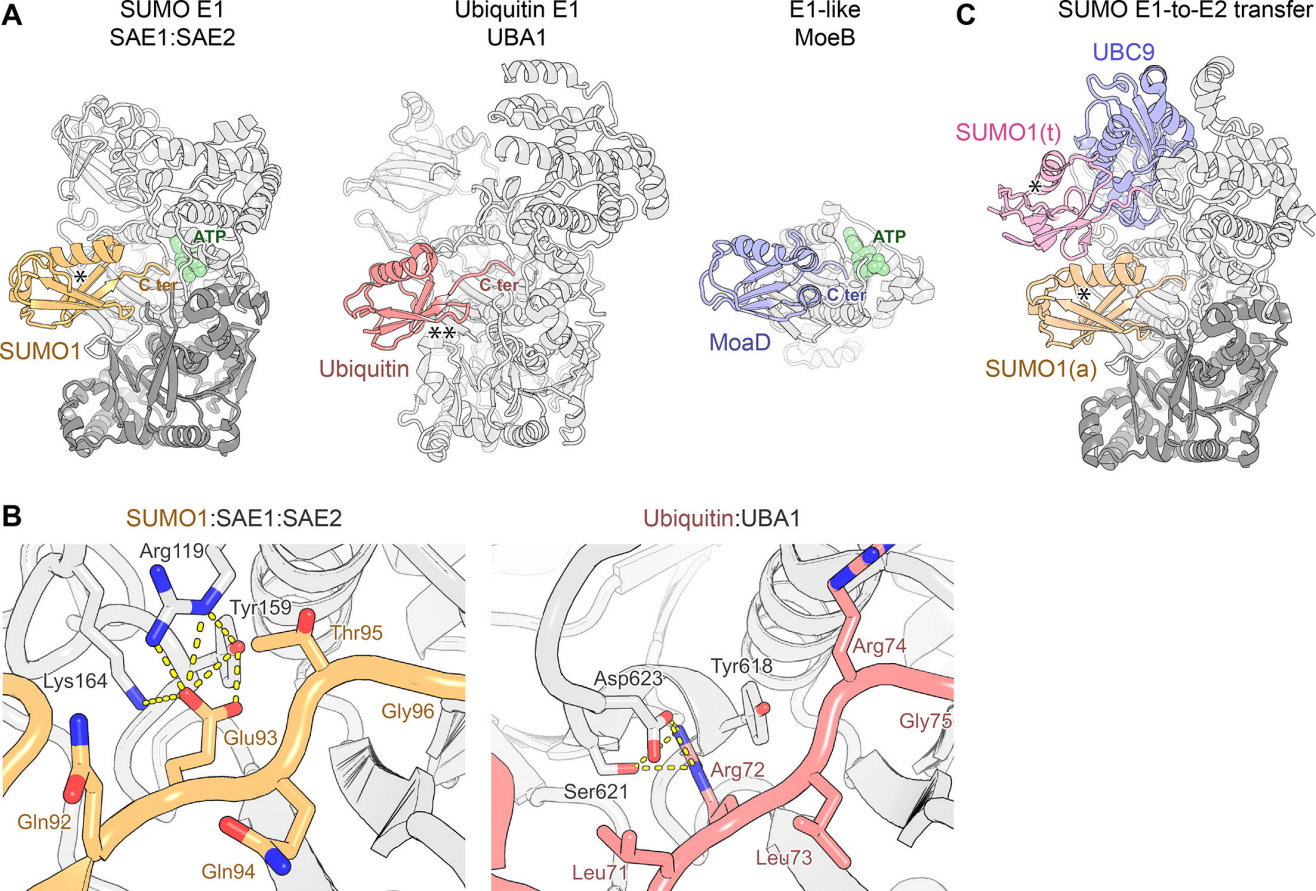
SUMO:E1 interaction compared with homologous complexes **A.** *Left:* Structure of the human heterodimeric SUMO-specific E1 enzyme composed of SAE1 and SAE2 subunits (coloured dark grey and light grey, respectively) in complex with SUMO1 (yellow), and ATP (green) (PDB 1Y8R). The SIM-binding groove – available for binding to a SIM – is marked with an asterisk (*). *Middle:* Structure of human ubiquitin-specific E1 enzyme UBA1 (light grey) in complex with ubiquitin (pink) (PDB 6DC6). The FCCH domain of UBA1, present in the PDB file, is hidden for easier comparison. The Ile44 patch of ubiquitin is marked with a double asterisk (**). *Right:* Structure of the prokaryotic E1-like MoeB enzyme (light grey) in compex with MoaD (blue) and ATP (green) (PDB 1JWA). A monomer is shown for easier comparison, although MoeB forms a C2-symmetric dimer. **B**. *Left:* A detail of the interaction between SUMO1 (light orange) and SAE1:SAE2 (light grey), centred on Glu93 of SUMO1, which is replaced by a Gln in SUMO2/3. Lys164 is acetylated in cells, leading to SAE1:SAE2’s preference for SUMO2 over SUMO1. PDB entry 1Y8R. *Right:* A corresponding detailed view of the interaction between ubiquitin (pink) and UBA1 (light grey), centred on Arg72, which is equivalent to Glu93 of SUMO1. PDB entry: 6DC6. In both views, selected side chains are shown as sticks and labelled and potential polar interactions of Glu93 of SUMO1/Arg72 of ubiquitin are indicated with dashed lines. **C**. Structure of the human SAE1:SAE2 complex bound to UBC9 and doubly loaded with SUMO. SUMO1(a) (yellow) represents a SUMO molecule that is undergoing adenylation and is equivalent to that in panel A, left. SUMO1(t) (pink) represents a SUMO molecule that is being transferred from Cys173 of SAE2 (light grey) to Cys93 of UBC9 (blue) (in fact, to obtain a stable mimetic, SUMO1(t) was attached to residue 129 of UBC9 and the two Cys residues were connected via a disulphide). The SIM-binding grooves – available for SIM binding in both SUMOs – are marked with an asterisk (*). PDB entry: 9DQB.

Comparing the crystal structures of SUMO-bound SAE1:SAE2 [28] with the ubiquitin-bound E1 UBA1 [105] and MoaD-bound MoeB [106] shows a similar orientation of SUMO, ubiquitin and MoaD relative to the structurally conserved active adenylation domain, highlighting the ancient character of this interaction (Figure 3A). All three UBL proteins contact the enzyme primarily through their β-sheet, including the surface corresponding to the Ile44 patch in ubiquitin, as well as through the C-terminus that protrudes towards the E1 active site, positioning the Gly–Gly motif near α-phosphate of ATP (Figure 2A and B).

Despite the conserved overall mode of binding, the individual contacts mediating these interactions differ, consistent with the specialisation of each enzyme for its cognate UBL. Discrimination probably depends on both the folded part of the modifier and its C-terminus. For example, in one critical area, Glu93 of SUMO1 is recognised by Arg119, Tyr159 and Lys164 of SAE2 [28] (Figure 3B, *left*). An Arg found at the equivalent position in ubiquitin would be incompatible with SAE2 binding; instead, in ubiquitin-specific UBA1, a basic residue equivalent to Arg119 is absent, Tyr618 (equivalent to SAE2’s Tyr159) interacts with ubiquitin Arg through a potential π:π interaction rather than an H-bond, and Lys164 is replaced by Asp623 in the equivalent position, making a salt bridge with the Arg residue (Figure 3B, *right*). This is a striking example of how a similar binding site can diverge to recognise markedly different sequences.

Interestingly, Glu93 of SUMO1 is replaced by Gln in SUMO2/3, and a recent study suggests that the acetylation of SAE2 on Lys164 results in the enzyme’s preference for SUMO2 over SUMO1 by favouring the recognition of Gln over Glu at this position [107], a mechanism that is consistent with the proximal location of Lys164 in structures (Figure 3B, *left*). Lys164 acetylation appears to change in a cell cycle-dependent manner, with its mitotic removal by the HDAC6 deacetylase reportedly triggering a wave of preferential SUMO1 modification of certain proteins.

Unlike Glu93, Thr95 of SUMO1 – which is again replaced by an arginine in ubiquitin – does not appear to be a specificity determinant, as the Thr95Arg mutant of SUMO1, sometimes used to permit trypsin cleavage upstream of the Gly–Gly motif, is well tolerated by SAE1:SAE2 [108].

The buried area of the SUMO:SAE2 interaction is considerable, exceeding 1000 Å^2^. The binding affinity of SAE1:SAE2 towards SUMO paralogues appears to be in the low micromolar range as reflected in both the dissociation constant (*Kd*) and Michaelis constant (*K*_M_) measurements [107,109].

Notably, SUMO is bound to the SAE2 adenylation domain in such a way that its characteristic SIM-binding groove remains accessible (Figures 3A and 2B). Some SUMO1:SAE1:SAE2 crystal structures show this groove engaged, either by a SIM located at the extreme C-terminus of SAE2, or, in another structure, partly by a fragment of the disordered FCCH domain originating from SAE1 [110]. These additional interactions might strengthen SUMO:E1 binding and might function to tether SUMO to the E1 enzyme during later stages when it is moving relative to it. In UBA1, an analogous extra contact with ubiquitin is formed by a very different, folded FCCH domain present in that enzyme [105] (not shown in Figure 3A).

## Interactions during the E1-to-E2 transfer

Following adenylation, SUMO is transferred to Cys173 of SAE2, forming a covalent SUMO∼SAE2 thioester intermediate. From there, SUMO is further transferred onto Cys93 of the SUMO-specific E2 enzyme UBC9, which is specifically recognised by the SUMO E1 complex through an extended interface [111,112]. These steps involve substantial movements of SUMO relative to the E1 enzyme and dramatic conformational changes within the SAE2 subunit of the E1 itself [112]. Furthermore, while one SUMO is being transferred onto UBC9, another SUMO can be adenylated, the two processes likely being allosterically coupled with each other, as suggested by observations in the ubiquitin system [113]. A recent cryogenic electron microscopy (cryoEM) structure of the stable mimetic of the doubly SUMO-loaded state of the SUMO E1 enzyme revealed that while the next SUMO is docked in the adenylation site described above, the SUMO that is transferred between SAE2 and UBC9 preferentially adopts a particular position – distinct from corresponding ubiquitin states – in which it leans against the face of UBC9 near Cys93 [112] (Figure 3C). This transient interaction – which buries a small surface area of less than 500 Å^2^ – involves a unique, largely polar SUMO surface that is proximal to, but distinct from, ubiquitin’s Ile44 patch. The interaction involves SUMO residues Asn60, Arg70, Gln94, among others. Interestingly, the SIM-binding groove again remains exposed and available for SIM binding.

## SUMO:E2 interactions

Beyond the transient interaction formed during the E1-to-E2 transfer, the SUMO-specific E2 enzyme UBC9 makes two distinct and critical non-covalent contacts with SUMO molecules. First, in addition to the covalent thioester bond with the donor SUMO, UBC9 forms non-covalent interactions that help stabilise this donor SUMO molecule when it is productively oriented for substrate transfer (the ‘closed’ conformation discussed below). These non-covalent interactions are not sufficient by themselves to result in a long-lived closed conformation, but they are nonetheless important. Second, UBC9 can simultaneously engage another SUMO molecule through a purely non-covalent interaction on the side opposite the active site, known as ‘backside’ (here called ‘back’).

### SUMO∼E2 thioester intermediate

**Figure 4:**
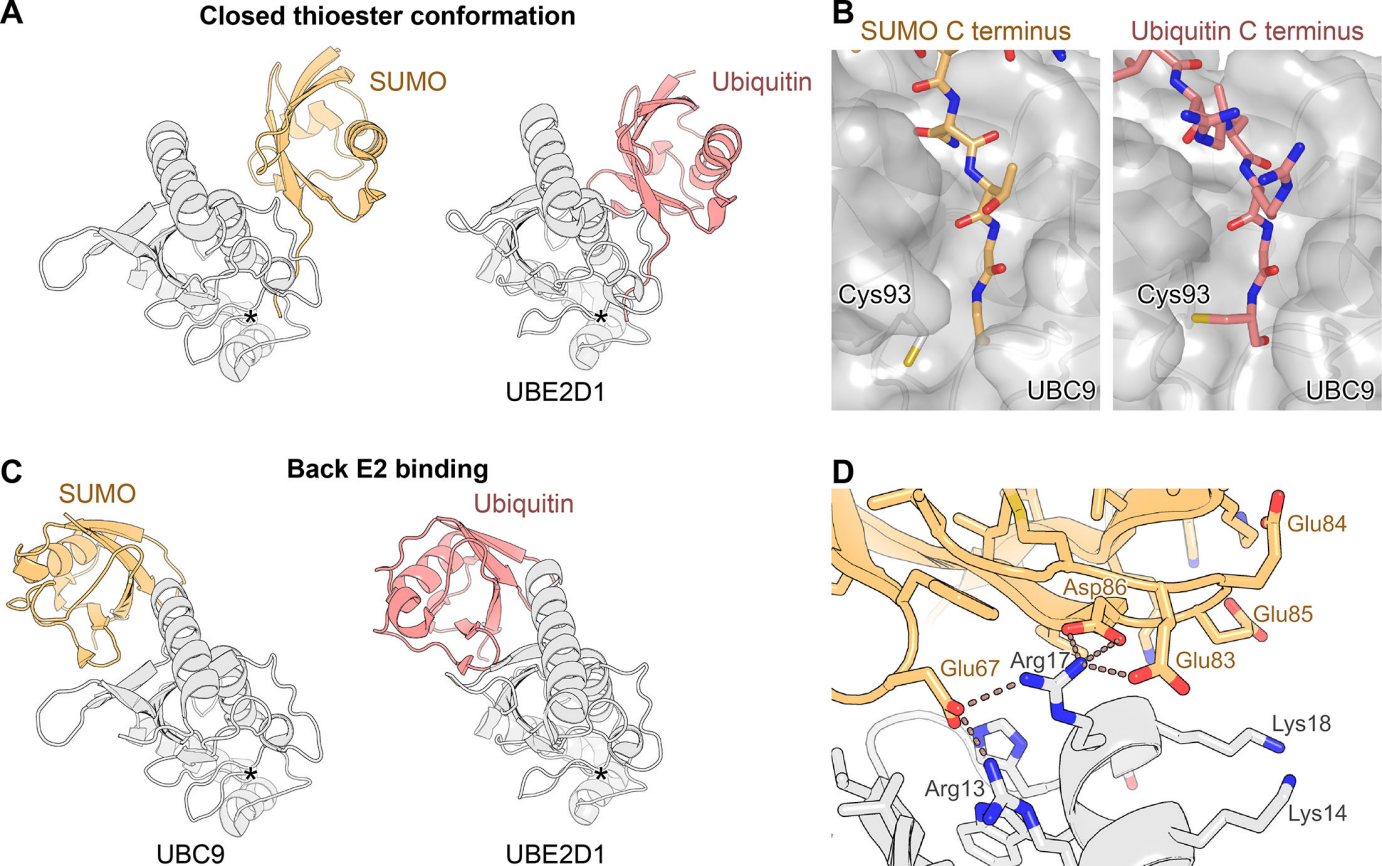
SUMO∼E2 thioester and SUMO:E2 back interaction **A.** Comparison of closed conformation adopted by SUMO∼UBC9 or ubiquitin∼UBE2D1 thioesters (more precisely, their stable mimetics), with SUMO/ubiquitin shown in yellow/blue and E2 enzymes in light grey. The mimetic of closed-conformation SUMO∼UBC9 was achieved using RANGAP1-linked SUMO1 in complex with UBC9 (RANGAP1 is not shown). PDB entries 3UIP and 4AP4, with E3 ligases omitted for clarity. **B**. *Left:* A zoomed-in fragment of mimetic of closed-conformation SUMO∼UBC9 from panel A showing the docking of the C-terminal region of SUMO1 on UBC9 (PDB 3UIP). In a real thioester, SUMO1 would be covalently bound to Cys93. *Right:* An equivalent view of a covalent thioester complex between UBC9 and a ubiquitin-derived peptide connected to Cys93 via a disulphide bond to mimic a thioester (PDB 6SYF). The two modifier tails adopt a similar conformation. **C**. Comparison between a non-covalent complex between human SUMO1 and UBC9 and an equivalent complex between human ubiquitin and a ubiquitin-specific E2 enzyme, UBED21 (UbcH5A), shown in the same orientation, with SUMO/ubiquitin shown in yellow/blue and E2 enzymes in light grey. PDB entries 2PE6 and 3PTF. **D**. A detail of the interaction between SUMO1 (light orange) and UBC9 (light grey), involving several negatively-charged residues on SUMO1 and several positively-charged ones on UBC9. Selected amino-acid side chains are shown as sticks, and some of them are labelled. PDB entry 2PE6.Black asterisks in **A** and **C** indicate the catalytic Cys residue of the indicated E2 enzymes, to which SUMO or ubiquitin is covalently attached in thioester intermediates.

The SUMO∼UBC9 thioester intermediate is of particular interest, as it is this short-lived molecule that donates the SUMO moiety to the substrate Lys residue in the final step of the enzymatic cascade (Figure 1B). UBC9 is not only a SUMO ‘carrier’ but also an enzyme that recognises and positions the target Lys residue and contributes to its deprotonation [29,114]. Like SUMO∼SAE2, the SUMO∼UBC9 intermediate appears relatively flexible, with widely different conformations captured in crystal structures [68,114]. Based on insights from the ubiquitin field [115–117], it is likely that SUMO, tethered to Cys93 of UBC9, retains substantial mobility and only intermittently samples a particular conformation relative to UBC9, called the ‘closed’ conformation, in which Gly97 of SUMO is well-oriented for a nucleophilic attack by the incoming Lys residue (Figure 4A). As a result, the final step of the SUMOylation reaction is slower than it could be if SUMO∼UBC9 were stably maintained in its closed conformation. As discussed later, this is precisely where SUMO E3 ligases come in, acting as scaffolds that stabilise SUMO∼UBC9 in the closed conformation, while simultaneously bringing it to a specific substrate or cellular location, resulting in local stimulation of SUMOylation.

To be stably adopted, the closed SUMO∼UBC9 conformation requires scaffolding by an E3 ligase, but it also relies on direct non-covalent contacts between SUMO and UBC9. This mechanism – the ‘deliberate’ inefficiency of a UBL∼E2 thioester intermediate, which makes space for regulation by an E3 ligase – has evolved before the divergence of SUMO and ubiquitin and is a shared feature of both systems. Also, the closed thioester conformations are largely superimposable in both cases, involving the Ile44 patch on ubiquitin [115,116] and a similar area on SUMO [68,86,88,118], among other regions (Figure 4A and Figure 2).

In the closed conformation, SUMO’s C-terminal region docks on UBC9 in a fairly defined conformation that helps present the Gly–Gly motif for nucleophilic attack [68] (Figure 4B). Therefore, sequence differences immediately upstream of this motif in ubiquitin might, if ubiquitin were thioester-linked to UBC9, be expected to perturb this docking and impair correct positioning. However, experimental data show that a short ubiquitin-derived C-terminal peptide chemically thioesterified to UBC9 is transferred to substrates even more efficiently than an equivalent SUMO-derived peptide, and an accompanying crystal structure of a similar peptide linked through a stable disulphide bond shows that the ubiquitin tail can be accommodated on UBC9 in a productive conformation that closely resembles that of the SUMO tail in the closed SUMO∼UBC9 conformation [119] (Figure 4B). It is even possible that the ubiquitin peptide binds better, adopting a closed-like conformation in a more constitutive manner. Despite differences from SUMO in sequence, steric clashes are prevented because the residues that are bulkier in ubiquitin (two Arg residues) correspond to residues that face outwards towards the solvent (Figure 4B, *right*). These observations suggest that discrimination against ubiquitin at the UBC9 stage is likely limited and, to the extent that it occurs, may depend more on the folded portion of SUMO/ubiquitin than on their C-terminus. More probably, the strict SUMO specificity is imposed earlier, during activation by the E1 enzyme, meaning that UBC9 is SUMO-specific primarily by virtue of its selective interaction with SAE1:SAE2 rather than with ubiquitin-specific E1 enzymes.

### Non-covalent SUMO:E2 back interaction

In addition to being covalently linked to a donor SUMO molecule via Cys93, UBC9 can use a surface on its opposite side, commonly referred to as the ‘backside’ (here we use ‘back’ to limit unfortunate connotations), to non-covalently bind a second SUMO [120–123]. Again, the back UBL interaction is a conserved feature of E2 enzymes observed also in the ubiquitin system [124]. Indeed, SUMO:UBC9 [122,123] and ubiquitin:E2 [125] crystal structures are very similar (Figure 4C). This is another SUMO interaction mediated by the β-sheet of SUMO/ubiquitin, including ubiquitin’s Ile44 patch, in this case with a limited interface area of ∼600 Å^2^ (Figure 2A and B). But as with the UBL:E1 interactions, the overall similar orientation of the bound UBL relative to the E2 masks differences in the specific underlying contacts, which vary between SUMO- and ubiquitin-specific complexes.

The SUMO:UBC9 back interaction is predominantly polar, involving multiple hydrogen bonds and salt bridges, with minimal estimated energetic contribution from buried hydrophobic residues (based on our PISA server analysis [122,123,126]. By contrast, the equivalent interaction between ubiquitin and a common E2 enzyme, UBE2D1 (UBCH5A) [125], is more hydrophobic, featuring few hydrogen bonds and no salt bridges. Furthermore, the affinity of the SUMO:UBC9 interaction – which has been reported to be in the low micromolar or even medium-to-high nanomolar range, possibly with some difference between paralogues [123,127,128] – is considerably higher than the mid- to high micromolar affinity typically observed between ubiquitin and its cognate E2 enzymes [129,130]. Due to its mostly polar character, the SUMO:UBC9 interaction may be particularly sensitive to salt concentration. Notably, an engineered SUMO2 variant with enhanced, nanomolar affinity for the back of UBC9 has been developed through a phage-display approach [128].

Among class II SUMO interactions, the SUMO:UBC9 back interaction – while still relying on a similar surface – is the most distinct in that it does not involve SUMO’s C-terminal tail but instead involves a negatively charged stretch of residues 83–86, which join Glu67 in interacting with a positively charged corner of UBC9 featuring Arg17 and Lys18 [122,123] (Figures 2A, B, 4D).

Functionally, the SUMO:UBC9 back interaction plays critical roles that remain incompletely elucidated. Although dispensable for mono- and di-SUMOylation, it is important for the elongation of SUMO chains [122,123]. Mechanistically, this may involve the recognition of an upstream SUMO unit of a growing chain to position UBC9’s active site near another SUMO unit further down the chain. Consistent with this role, the enhanced back-binding engineered SUMO2 mentioned above, devoid of the Gly–Gly motif (and hence not itself conjugatable), selectively inhibits poly- but not monoSUMOylation in cells [128]. More generally, the back interaction may help recruit UBC9 to substrates or subcellular locations that are already SUMOylated, establishing a positive feedback loop that promotes further SUMOylation. Furthermore, the SUMO:UBC9 interaction supports the binding of many SUMO E3 ligases, which often use the back-bound SUMO as an anchoring point to stabilise the SUMO∼UBC9 complex in its closed conformation.

## SUMO:protease interactions

Another class of enzymes that form key non-covalent interactions are SUMO-specific proteases, which recognise SUMO’s fold and C-terminus to catalyse specific cleavage after the Gly–Gly motif. At present, the strongest evidence for specific recognition and processing of SUMO – including structural studies – is available for SENPs/ULPs [27,30,67,131] and USPL1 [71].

Although SENPs and USPL1 are structurally unrelated, they engage partially overlapping surfaces on SUMO, again including the β-sheet region (encompassing the equivalent of the Ile44 patch of ubiquitin) and the C-terminal tail (approximately residues 60–75 and 89–97 in SUMO:SENP and additionally 28–33 in SUMO:USPL1) [27,30,67,71,131] (Figure 2A and B). The C-terminus becomes stabilised, in SENPs/ULPs – but not in USPL1 – partly adopting a β-strand conformation [12,27,30,67,71]. All these interactions together form an extensive binding interface exceeding 1000 Å² in both protease families. For SENP1, the *Kd* is in the high nanomolar range for immature SUMOs and around 10 nM for both SUMOylated proteins and mature SUMOs [67]. The SUMO:SENP interaction is similar to that between NEDD8 and SENP8 [96], whereas the SUMO:USPL1 complex resembles those between ubiquitin and canonical USP proteases [132].

Although all SUMO paralogues interact with SENP proteases in a broadly similar way, SENPs have partly different residues in their SUMO-binding sites, and so do the SENP-binding sites on SUMOs, explaining why some SENPs prefer particular SUMO paralogues. For example, a basic patch around residue Arg624 of SENP5 enables an electrostatic interaction with residue Asp63 of SUMO2/3, which appears less favourable in the case of SUMO1, where the equivalent residue is Glu67 [133]. For SENP6 and SENP7, a preference for SUMO2/3 seems to be partly due to the unique presence of a loop in the N-terminal part of the SENP catalytic domain, which establishes specific interactions with the residues Arg61, Pro66 and Asn68 of SUMO2/3 [134]. The non-SENP protease USPL1 also prefers SUMO2/3 over SUMO1, both in binding and in catalytic activity, apparently due to specific recognition of the C-terminal region of SUMO2/3 [71].

Notably, in both SUMO:SENP and SUMO:USPL1 complexes, the SIM-binding groove on SUMO remains accessible for potential additional interactions [27,30,67,71,131] (Figure 2A and B). Fittingly, SENP family proteases harbour extended, largely intrinsically disordered regions harbouring SIM motifs [91,92,135–137]. Thus, while the primary interaction with SUMO is mediated by the catalytic domain as mentioned above, SENPs may use SIMs to further recognise and position their substrates. One example is the presence of multiple SIMs in the extensions of yeast Ulp2, human SENP6 and human SENP7, which have been implicated in the preferential recognition of polySUMOylated substrates by these proteases [135–137].

## Convergent and distinct contacts in SUMO:enzyme interactions

As is evident in Figure 2, the different non-covalent interactions described in this review (with E1, E2 and proteases) – despite their distinct evolutionary origins – tend to involve a similar surface on SUMO, overlapping with the Ile44 patch on ubiquitin. This seems to have been the primary site attracting new interactions in an ancestral UBL protein, and although, as we have argued, this role seems to have been largely taken over, in SUMO, by the hydrophobic SIM-binding groove, SUMO has preserved the use of this surface for binding to these key enzymes.

**Figure 5:**
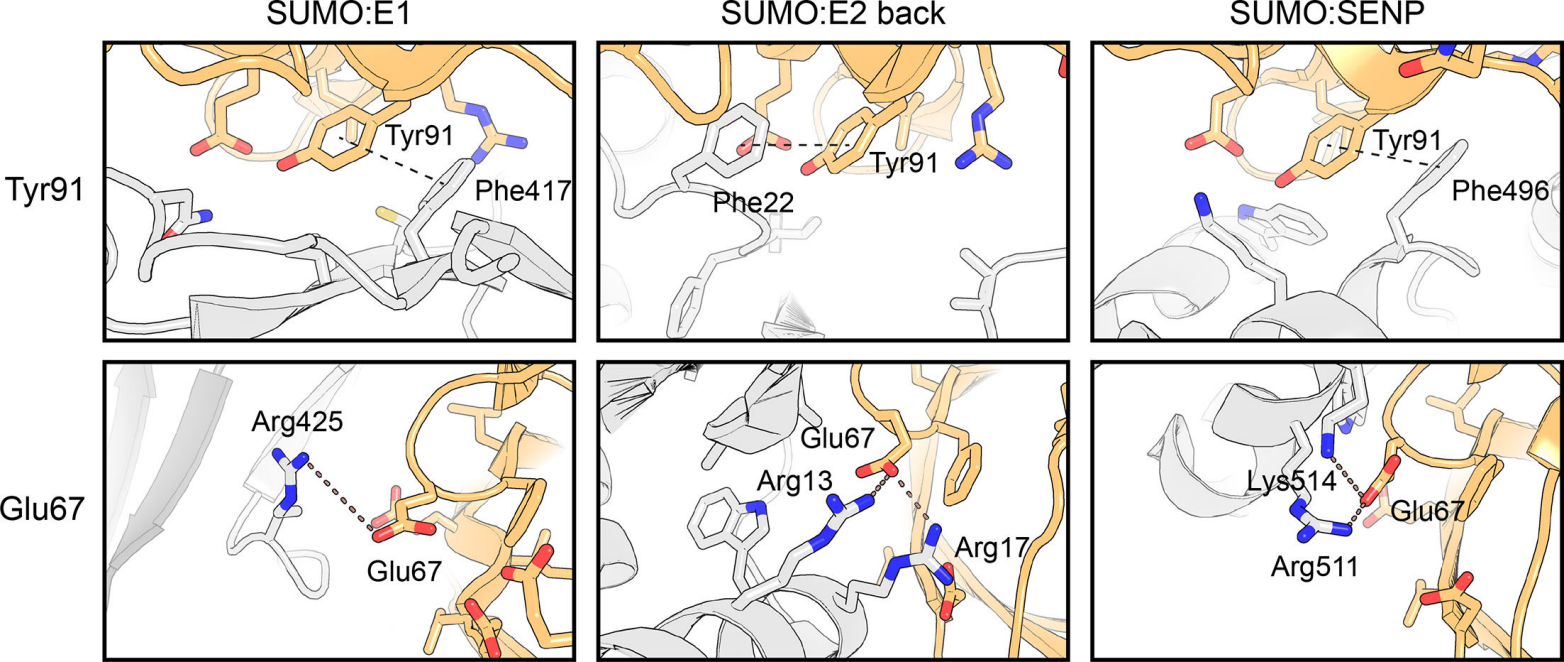
Convergent evolution of contacts between SUMO and different enzymes Examples of contacts made by Tyr91 (*top*) and Glu67 (*bottom*) of SUMO1 with residues on the SUMO E1 enzyme subunit SAE2 (*left*), the back-binding site on SUMO E2 enzyme UBC9 (*centre*), and SUMO-specific protease SENP1 (*right*). SUMO1 is always shown in light orange and enzymes in light grey. Selected amino-acid side chains are shown as sticks, and possible inter-residue contacts are indicated with dashed lines. Tyr91 tends to form π:π interactions with Phe residues, whereas Glu67 shows a propensity to make salt bridges or electrostatic interactions with Arg residues. PDB entries 1Y8R (SUMO1:E1 complex), 2PE6 (SUMO1:UBC9 back) and 2IY1 (SUMO1:SENP1).

The interactions between SUMO and its key enzymes reflect both divergent and convergent evolutionary processes. They illustrate divergence because – although they structurally resemble the equivalent interactions formed by ubiquitin and other UBL proteins – the detailed contacts differ substantially, ensuring that the enzymes remain specific for SUMO, sometimes preferring a particular SUMO paralogue. At the same time, these interactions exemplify convergent evolution, since the different enzymes – E1, E2 and proteases – while coevolving with SUMO, have independently converged on certain analogous ways of engaging their cognate modifier in such a way that it allows discriminating against other UBLs. The best example is π:π contacts with Tyr91 (Phe in SUMO2/3) – a hotspot involved in every single enzyme interactions discussed here (Figure 2) – formed by aromatic residues on some of the described enzymes, including Phe417 on SAE2, Phe22 on UBC9 (back interaction), and Phe496 on SENP1 (Figure 5A). Another example is provided by Glu67 of SUMO1 (Asp in SUMO2/3), which forms salt bridges or electrostatic interactions with Arg or Lys residues in the cognate enzymes, including Arg13 and Arg17 in UBC9 (back interaction), Arg511 and Lys514 in SENP1, and possibly – although the distance seems larger – Arg425 in SAE2 (Figure 5B). Neither Tyr91 nor Glu67 is conserved in ubiquitin, marking them as signature hotspots of SUMO.

While further individual examples of convergent contacts could be mentioned, it should be stressed that most contacts remain nonetheless distinct in each case, demonstrating how an overlapping surface on SUMO can be used for biochemically distinct interactions.

## Summary and outlook

For their small size, SUMO proteins form an impressive number of different non-covalent protein:protein interactions, some of which are critical to their function as protein modifiers, either by allowing the modification (or demodification) to occur in the first place or by mediating recognition of SUMOylation signals to trigger downstream effects. In this review, we aimed to summarise what is known about the non-covalent interactions between SUMO and the core enzymatic machinery of the SUMOylation cycle (Figure 1B). Together with the accompanying review dedicated to the interactions with SUMO E3 ligases and downstream effectors, this discussion provides a comprehensive overview of SUMO as a non-covalent interaction hub.

All SUMO:enzyme interactions described here involve structured protein domains that – despite making diverse specific contacts – consistently engage a similar surface on SUMO, corresponding to the opposite side of its β-sheet with respect to the SIM-binding groove (Figure 2). These interactions could, therefore, be broadly classified as class II SUMO interactions. They are mutually exclusive sterically with one another, which is consistent with each enzyme catalysing a distinct step in SUMO’s life cycle. The interactions with E1, E2 and USPL1 are homologous to those between ubiquitin and its cognate equivalent enzymes, but the underlying specific contacts are different in SUMO and ubiquitin-specific complexes, reflecting a long history of divergent evolution producing orthogonal enzymatic systems.

The separation between the ubiquitin and SUMO systems already existed in the last common eukaryotic ancestor [33], but we lack information about the earlier evolutionary stages when the two systems became distinct and specialised following the apparent duplication of a single system. New insights into this question might come from studies of ancestral prokaryotic systems – several of which have recently been discovered but, so far, none of them sharing the distinct features of SUMO such as the SIM-binding groove [36,74–82] – or from bioinformatic ancestral sequence reconstruction approaches.

Although the core architecture of the key SUMO:enzyme interactions was elucidated a few decades ago, we do not always have detailed information about the distinct stages of these enzymatic processes and the, sometimes subtle, conformational changes and allosteric signals that are likely to drive the transitions between them and ensure directionality and co-ordination. A preview of these kinds of insights has been provided by the recent high-resolution cryoEM studies on the co-ordination between E1 and E2 enzymes in the ubiquitin [113] and SUMO [112] pathways, and we believe that cryoEM, aided by novel computational approaches, will likely continue to provide complementary insights to the seminal X-ray crystallography-based studies obtained in the previous decades.

Each of the described SUMO:enzyme interactions is sterically compatible with a simultaneous SUMO:SIM interaction – a hallmark of the SUMO signalling system, absent from the ubiquitin realm. This has important mechanistic consequences. For example, the E1 subunit SAE2 and SENP proteases contain flexible extensions that harbour SIM motifs, and these SIMs could make additional interactions with the SUMO molecules engaged by catalytic domains of these proteins – as [91,110,138]. Moreover, as discussed in more detail in the accompanying review, the SUMO molecules interacting with the E2 enzyme UBC9 can simultaneously bind to SIMs belonging to a SUMO E3 ligase, which thus stabilises the conjugation machinery in a productive conformation. How exactly are class II interactions co-ordinated with SIM-mediated class-I interactions? This is another outstanding question in the field.

Finally, it remains unclear to what extent the various possible SUMO signals – arising from different paralogues, chain lengths and topologies, combinations with other UBLs, et cetera – play distinct functional roles. If they do, an additional question is how such signals are balanced given their regulation by largely shared enzymatic machinery. A recent report that SUMO E1 may prefer SUMO1 or SUMO2/3 depending on the acetylation status of the SAE2 Lys164 residue provides one emerging mechanism [107], as do observations that certain SUMO-specific proteases favour particular paralogues or chains over monoSUMOylation [71,97,133], but further mechanisms are likely to remain undiscovered.

Owing to the seminal structural biology and mechanistic biochemistry work on the discussed SUMOylation enzymes and related UBL enzymes, we have a good understanding of the key non-covalent events controlling the SUMOylation cycle. The current challenge is to elucidate how the different key events are co-ordinated with one another – both at the molecular level and within the spatially compartmentalised environment of the cell – in such a way that directionality, specificity and an appropriate level of signalling output are achieved.

**Summary**

- Protein SUMOylation is an essential process for the development and viability of eukaryotic organisms.
- Small ubiquitin-like modifier (SUMO)’s maturation, conjugation and deconjugation depend on specific non-covalent interactions between SUMO and its cognate enzymes.
- SUMO-specific enzymes discriminate between SUMO and ubiquitin by recognising differences in their surface and C-termini; some enzymes have preferences for particular SUMO paralogues. The specificity of the E1–E2 conjugation cascade for SUMO over ubiquitin is determined mainly at the E1 level.
- The core interactions between SUMO and E1, E2 and protease enzymes involve a similar surface on SUMO located on the side opposite the SUMO-interacting motif (SIM)-binding groove and overlapping with the Ile44 patch on ubiquitin. This makes the listed SUMO:enzyme interactions mutually exclusive while allowing compatibility with simultaneous SIM binding.
- Future work should elucidate the mechanisms through which the various enzymatic processes are co-ordinated to achieve specificity and a correct level of modification in the cell.

## Open Access

This article has been published open access under our Subscribe to Open programme, made possible through the support of our subscribing institutions, learn more here: https://portlandpress.com/pages/open_access_options_and_prices#conditional

## Abbreviations

AIM: atg8-interacting motif
AMP: adenosine monophosphate
ATG8: autophagy-related protein 8
ATP: adenosine triphosphate
CUL1: cullin 1
DEN1: deNEDDylase 1
DUB: Deubiquitylating enzyme
E1: UBL activating enzyme
E2: UBL conjugating enzyme
E3: UBL ligase
ESC2: establishment of sister chromatid cohesion protein 2
FANCI: fanconi anemia complementation group I protein
FAS: cell surface death receptor
FCCH: first catalytic cysteine half-domain
HDAC6: histone deacetylase 6
ISG15: interferon-stimulated gene 15
LC3: 1b-light chain 3
LIR: LC3-interacting region
NEDD8: neural precursor cell expressed, developmentally down-regulated protein 8
NEDP1: NEDD8-specific protease 1
NFATC2IP: nuclear factor of activated t-cells, cytoplasmic 2–interacting protein
NIP45: nuclear interactor of protein 45
PDB: Protein Data Bank
PML: promyelocytic leukemia protein
PTM: post-translational modification
RAD51 and RAD52: dna repair proteins 51 and 52
RANBP2: RAN-binding protein 2
RANGAP1: ran gtpase-activating protein 1
RARA: retinoic acid receptor alpha
RING: really interesting new gene
RNA: ribonucleic acid
SAE1 and SAE2: SUMO-activating enzyme 1 and 2
SAMP: small archaeal modifier protein
SENP: sentrin/SUMO-specific protease
SIMs: SUMO-interacting motifs
SUMO: small ubiquitin-like modifier
TNFR1: tumor necrosis factor receptor 1
UAF1: USP1-associated factor 1
UBA1: ubiquitin-activating enzyme 1
UBA5: UFM1-activating enzyme
UBC9: ubiquitin-conjugating enzyme-family 9 (a SUMO-specific E2 enzyme)
UBL: ubiquitin-like
UFM1: ubiquitin-fold modifier 1
ULP: ubiquitin-like protease
USPL1: Ubiquitin-specific peptidase-like 1
ZNF451: zinc finger protein 451
cryoEM: cryogenic electron microscopy
